# The genome sequence of the Blue Long-horned Snailkiller, *Sepedon sphegea* (Fabricius, 1775) (Diptera: Sciomyzidae)

**DOI:** 10.12688/wellcomeopenres.28240.1

**Published:** 2026-10-28

**Authors:** Steven Falk, Ryan Mitchell, Olga Sivell, Duncan Sivell, Rachel Lennon, University of Oxford and Wytham Woods Genome Acquisition Lab, Natural History Museum Genome Acquisition Lab, Darwin Tree of Life Barcoding Collective, Wellcome Sanger Institute Tree of Life Management, Samples and Laboratory team, Wellcome Sanger Institute Scientific Operations: Sequencing Operations, Wellcome Sanger Institute Tree of Life Core Informatics team, Tree of Life Core Informatics collective, Darwin Tree of Life Consortium

**Affiliations:** 1 Independent researcher, Kenilworth, Warwickshire, England, UK; 2 Independent researcher, Sligo, Sligo County, Ireland; 3 Natural History Museum, London, England, UK; 4 School of Biodiversity, One Health and Veterinary Medicine, University of Glasgow, Glasgow, Scotland, UK

**Keywords:** Sepedon sphegea, Blue Long-horned Snailkiller, genome sequence, chromosomal, Diptera

## Abstract

We present a genome assembly from an individual male *Sepedon sphegea* (Blue Long-horned Snailkiller; Arthropoda; Insecta; Diptera; Sciomyzidae). The primary assembly has a total length of 747.79 megabases. Most of the primary assembly (98.04%) is scaffolded into 7 chromosomal pseudomolecules, including the X and Y sex chromosomes. The mitochondrial genome has also been assembled, with a length of 19.16 kilobases. This assembly was generated as part of the Darwin Tree of Life project, which produces genomes for eukaryotic species found in Britain and Ireland.

## Species taxonomy

Eukaryota; Opisthokonta; Metazoa; Eumetazoa; Bilateria; Protostomia; Ecdysozoa; Panarthropoda; Arthropoda; Mandibulata; Pancrustacea; Altocrustacea; Allotriocarida; Hexapoda; Insecta; Dicondylia; Pterygota; Neoptera; Eumetabola; Endopterygota; Aparaglossata; Panorpida; Diptera; Brachycera; Muscomorpha; Eremoneura; Cyclorrhapha; Schizophora; Acalyptratae; Sciomyzoidea; Sciomyzidae; *Sepedon*; *Sepedon sphegea* (Fabricius, 1775) (NCBI:txid1226606)

## Background

*Sepedon sphegea* (Fabricius, 1775), the Blue Long-horned Snailkiller, is a sciomyzid fly found across the Palaearctic region ( Khaghaninia *et al.*, 2018). Adults are 8–11 mm long, with a bluish-black body, elongated orange-red femurs and no black pruinose parafrontal spots ( Knutson & Orth, 1984). The species belongs to a group of three similar species that are difficult to distinguish. Its even, apically rounded cercus and lack of an anteroventral projection distinguish it from *S. aenescens* and *S. femorata* ( Knutson & Orth, 1984).

*S. sphegea* occurs in undisturbed habitats, including marshland, wet meadows, canals and pond margins ( Knutson *et al.*, 1973). It is multivoltine, with adults that overwinter and may be observed throughout the year, most often during the March to October flight period in the UK ( Gaponov, 2016; Neff & Berg, 1966).

The predatory larvae of this family feed at the water surface on aquatic snails and opportunistically on other molluscs, giving rise to the name ‘snail-killing flies’ ( Berg, 1973; Rozkošný, 1987). These mollusc-eating larvae have attracted interest as potential biological control agents against agricultural pest snails and slugs, and against snails involved in the transmission of schistosomiasis to humans ( Khaghaninia *et al.*, 2016; Tirgari, 1977).

We present a chromosome-level genome sequence for *Sepedon sphegea*, produced using the Tree of Life pipeline. The sequence is based on a specimen collected from Wytham Woods, Oxfordshire, UK (Figure 1). This assembly was generated as part of the Darwin Tree of Life Project, which aims to generate high-quality reference genomes for all named eukaryotic species in Britain and Ireland to support research, conservation, and the sustainable use of biodiversity ( Darwin Tree of Life Project Consortium, 2022).

**Figure 1.**
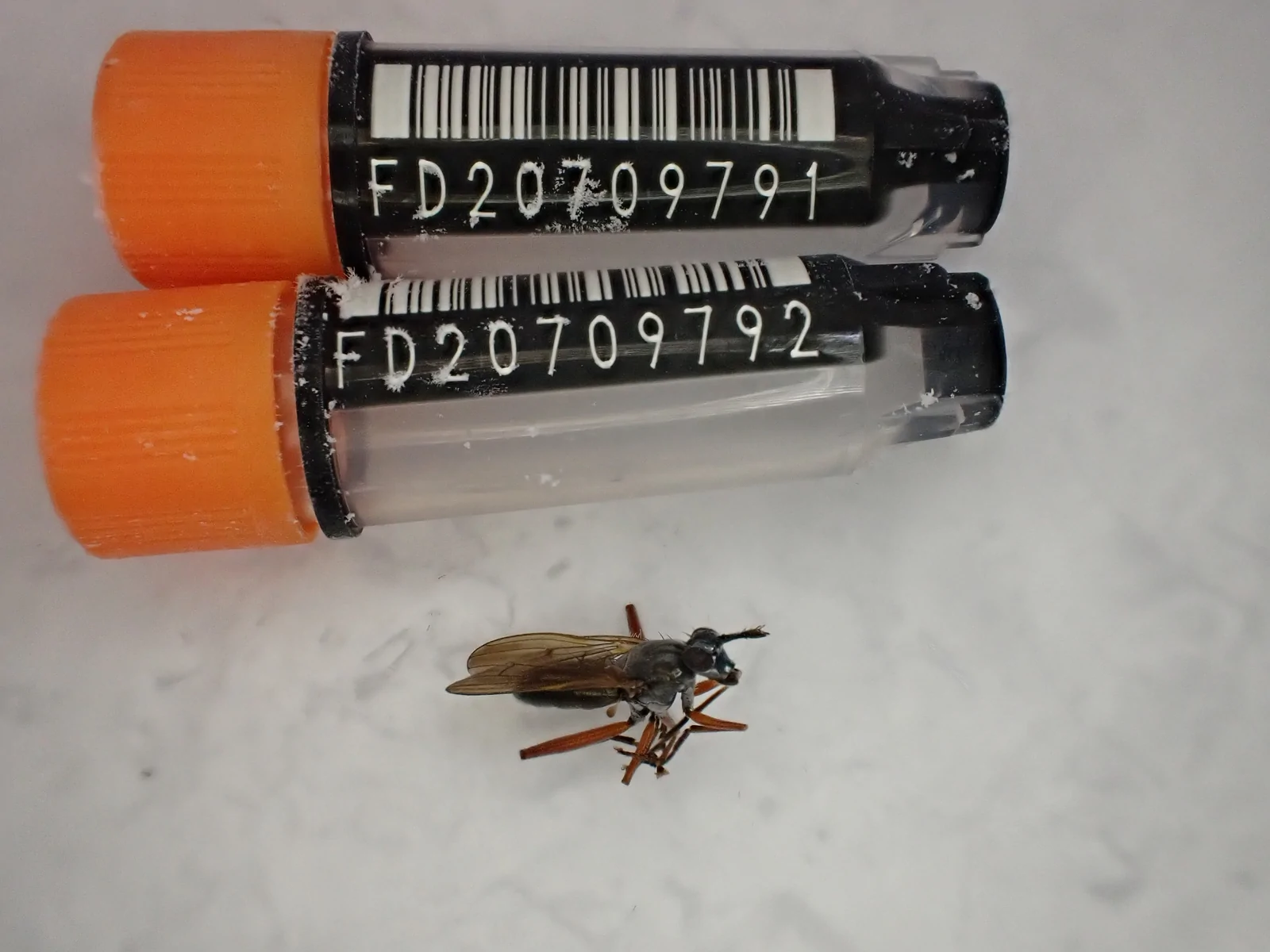
The *S. sphegea* specimen (Ox001307, idSepSphe2) used for genome sequencing.

## Methods

### Sample acquisition and DNA barcoding

Two adult *Sepedon sphegea* specimens were used: a male specimen for genome and RNA sequencing (specimen ID Ox001307, ToLID idSepSphe2; Figure 1) and a specimen for Hi-C sequencing (specimen ID NHMUK014446900, ToLID idSepSphe1). The specimen used for genome and RNA sequencing was collected from Wytham Woods, Oxfordshire, UK (latitude 51.764, longitude −1.337) on 2021-04-23 by Steven Falk, who also identified it. The specimen used for Hi-C sequencing was collected from Cothill Fen National Nature Reserve, England, UK (latitude 51.693, longitude −1.336) by Ryan Mitchell and Olga Sivell and identified by Duncan Sivell. Sample metadata were collected in line with the Darwin Tree of Life project standards described by Lawniczak *et al*. (2022).

The initial identification was verified by an additional DNA barcoding process according to the framework developed by Twyford *et al.*. (2024). A small sample was dissected from the specimen and stored in ethanol, while the remaining parts were shipped on dry ice to the Wellcome Sanger Institute (WSI) (see the protocol). The tissue was lysed, the COI marker region was amplified by PCR, and amplicons were sequenced and compared to the BOLD database, confirming the species identification ( Crowley *et al.* , 2023). Following whole-genome sequencing, the relevant DNA barcode region was also used alongside the initial barcoding data for sample tracking at the WSI ( Twyford *et al.* , 2024). The standard operating procedures for Darwin Tree of Life barcoding are available on protocols.io.

### Nucleic acid extraction

High molecular weight (HMW) DNA extraction protocols developed by the Tree of Life Core Laboratory at the Wellcome Sanger Institute (WSI) are available on protocols.io ( Howard *et al.* , 2025). Tissue from the idSepSphe2 sample was weighed and triaged to select the appropriate extraction workflow.

For library DTOL13251668, 8.3 mg of head and thorax tissue was homogenised by PowerMash using a PowerMasher II tissue disruptor. HMW DNA was extracted in the WSI Scientific Operations core using the Manual MagAttract protocol. DNA was sheared into an average fragment size of 12–20 kb following the Diagenode Megaruptor3 for PacBio HiFi Sheared DNA was purified by manual SPRI (solid-phase reversible immobilisation).

The concentration of the sheared and purified DNA was assessed with a NanoDrop spectrophotometer and a Qubit Fluorometer using the Qubit dsDNA High Sensitivity Assay kit. Fragment size distribution was evaluated by running the sample on the Femto Pulse system. For this sample, post-shearing DNA measurements included a Qubit concentration of 21.8 ng/μL, an average post-shearing fragment size of 13.6 kb and a yield of 1,199.00 ng.

RNA was extracted from abdomen tissue of the idSepSphe2 specimen in the Tree of Life Laboratory at the WSI using the RNA Extraction: Automated MagMax™ *mir*Vana protocol. The RNA concentration was assessed with a NanoDrop spectrophotometer and a Qubit Fluorometer using the Qubit RNA Broad-Range Assay kit. RNA integrity was assessed using the Agilent RNA 6000 Pico Kit and Eukaryotic Total RNA assay.

### PacBio HiFi library preparation and sequencing

Library DTOL13251668 was prepared with the SMRTbell Prep Kit 3.0 (Pacific Biosciences) according to the manufacturer’s instructions. The kit includes reagents for end repair/A-tailing, adapter ligation, post-ligation SMRTbell bead clean-up, and nuclease treatment. Size selection and clean-up were performed using diluted AMPure PB beads (Pacific Biosciences). DNA concentration was quantified with a Qubit Fluorometer v4.0 (Thermo Fisher Scientific) and the Qubit 1X dsDNA HS assay kit. Final library fragment size was assessed with the Agilent Femto Pulse Automated Pulsed Field CE Instrument (Agilent Technologies) using the gDNA 55 kb BAC analysis kit.

The library was sequenced using the Sequel II system (Pacific Biosciences, California, USA). Library loading concentrations were in the range 40–135 pM. SMRT Link software (Pacific Biosciences) was used to configure and monitor the run and to perform primary and secondary data analysis.

### Hi-C

***Sample preparation and crosslinking***

Hi-C data were generated from the idSepSphe1 head and thorax sample using the Arima-HiC v2 kit (Arima Genomics). Following the manufacturer’s instructions, tissue was fixed and DNA crosslinked using TC buffer to a final formaldehyde concentration of 2%. The tissue was homogenised using the Diagnocine Power Masher-II. Crosslinked DNA was digested with a restriction enzyme master mix, biotinylated, and ligated. Clean-up was performed with SPRISelect beads before library preparation. DNA concentration was measured with the Qubit Fluorometer (Thermo Fisher Scientific) and Qubit HS Assay Kit. The biotinylation percentage was estimated using the Arima-HiC v2 QC beads.

***Hi-C library preparation and sequencing***

Biotinylated DNA constructs were fragmented using a Covaris E220 sonicator and size-selected to 400–600 bp using SPRISelect beads. DNA was enriched with Arima-HiC v2 kit Enrichment beads. End repair, A-tailing, and adapter ligation were carried out with the NEBNext Ultra II DNA Library Prep Kit (New England Biolabs), following a modified protocol where library preparation occurs while DNA remains bound to the Enrichment beads. Libraries were amplified with KAPA HiFi HotStart mix and a custom Unique Dual Index (UDI) barcode set (Integrated DNA Technologies). Depending on sample concentration and biotinylation percentage determined at the crosslinking stage, libraries were amplified with 10–16 PCR cycles. Post-PCR clean-up was performed with SPRISelect beads. Libraries were quantified using the AccuClear Ultra High Sensitivity dsDNA Standards Assay Kit (Biotium) and a FLUOstar Omega plate reader (BMG Labtech).

Prior to sequencing, libraries were normalised to 10 ng/μL. Normalised libraries were quantified again to create equimolar and/or weighted 2.8 nM pools. Pool concentrations were checked using the Agilent 4200 TapeStation (Agilent) with High Sensitivity D500 reagents before sequencing. Libraries were sequenced on the Illumina NovaSeq 6000, generating paired-end 150-bp reads.

### RNA library preparation and sequencing

Libraries were prepared with the NEBNext® Ultra II Directional RNA Library Prep Kit for Illumina (New England Biolabs), following the manufacturer’s instructions. Poly(A) mRNA was isolated from 100 ng of total RNA using oligo(dT) beads, converted to cDNA, and uniquely indexed; 14 PCR cycles were performed. Libraries were prepared with an intended average fragment size of 100–300 bp. Libraries were quantified, normalised, and pooled equimolarly to a final concentration of 2.8 nM before dilution to 150 pM for loading. Sequencing was carried out on the Illumina NovaSeq X, generating paired-end reads.

### Genome assembly

Prior to assembly of the PacBio HiFi reads, a database of *k*-mer counts ( *k* = 31) was generated from the filtered reads using FastK. The *k*-mer frequency distribution was analysed with GenomeScope 2.0 ( Ranallo-Benavidez *et al.*, 2020), providing fitted point estimates of haploid genome size, heterozygosity and repeat content.

The HiFi reads were assembled using Hifiasm ( Cheng *et al.*, 2021) with the --primary option. Haplotypic duplications were identified and removed using purge_dups ( Guan *et al.*, 2020). The Hi-C reads ( Rao *et al.* , 2014) were mapped to contigs using bwa-mem2 ( Vasimuddin *et al.*, 2019), and the contigs were scaffolded in YaHS ( Zhou *et al.*, 2023) with the --break option for handling potential misassemblies. The scaffolded assemblies were evaluated using Gfastats ( Formenti *et al.* , 2022), BUSCO ( Tegenfeldt *et al.*, 2025) and MerquryFK ( Rhie *et al.*, 2020).

The mitochondrial genome was assembled using MitoHiFi ( Uliano-Silva *et al.* , 2023). The MitoHiFi reference was the mitochondrial genome of *Trypetoptera punctulata* (MK644823.1).

### Assembly curation

The assembly was decontaminated using the Assembly Screen for Cobionts and Contaminants ( ASCC) pipeline. The flat files and maps for curation were generated with TreeVal. Manual curation was conducted primarily in PretextView and HiGlass ( Kerpedjiev *et al.* , 2018). Scaffolds were visually inspected and corrected as described by Howe *et al*. (2021). The primary and alternate assemblies were combined for curation. Manual corrections included 86 breaks and 266 joins. This reduced the scaffold count by 54.7%, increased the scaffold N50 by 57.0%, and increased the total assembly length by 2.3%. The curation process is described at sanger-tol/curation-resources. A Hi-C contact map of the primary assembly was generated with PretextSnapshot.

### Assembly quality assessment

The MerquryFK tool ( Rhie *et al.*, 2020) was run in a Singularity container ( Kurtzer *et al.*, 2017) to evaluate *k*-mer completeness and assembly quality for the primary and alternate haplotypes using the *k*-mer database ( *k* = 31) computed prior to genome assembly. The analysis outputs included assembly QV scores and completeness statistics.

Gene-set completeness was assessed with BUSCO ( Tegenfeldt *et al.*, 2025), using the brachycera_odb12 lineage dataset ( *n* = 5,912) in eukaryotic genome mode, with miniprot ( Li, 2023). This assessment was produced by sanger-tol/blobtoolkit.

The primary assembly was assessed using BlobToolKit ( Challis *et al.*, 2020). PacBio reads were mapped to the assembly to derive sequence coverage. Within this workflow, BUSCO ( Tegenfeldt *et al.*, 2025) was used to assess gene-set completeness. DIAMOND BLASTp searches of extracted BUSCO proteins and DIAMOND BLASTx searches of assembly sequences against UniProt Reference Proteomes ( Buchfink *et al.*, 2021), followed by BLASTn searches of sequences without DIAMOND BLASTx hits against the NCBI nt database ( Altschul *et al.*, 1990), supported taxonomic assignment. Coverage, sequence composition, taxonomic assignment and the workflow’s BUSCO results were integrated into a BlobDir for interactive inspection and visualisation. The snail and blob plots were rendered locally from this hosted BlobDir using BlobTk. The scaffold-length radial scale function for the snail plot was set to linear ( Challis & Blaxter, 2026). The blob plot used circular markers with area proportional to scaffold length ( Challis *et al.*, 2020).

Ancestral linkage groups were painted along the chromosome-level scaffolds using *busco-alg-painter*. Complete BUSCO genes (single-copy and duplicated) from the diptera_odb12 full table were assigned to the 10 Brachycera ancestral linkage groups (db1a–db4b, db5 and db6) described by Gries *et al.*. (2026). Chromosome identifiers and lengths were obtained from NCBI Datasets for GCA_988143715.1, and BUSCO positions were plotted along chromosomes drawn to scale.

## Genome sequence report

### Sequence data

PacBio sequencing of the *S. sphegea* specimen generated 28.84 Gb (gigabases) from 2.53 million reads. These reads were used to assemble the genome. GenomeScope 2.0 analysis using a *p* = 2 model gave a *k*-mer-based haploid genome size estimate of 645.59 Mb, with heterozygosity of 1.95% and repeat content of 45.40%; the full-model fit was 96.56% (Figure 2). The GenomeScope 2.0 estimates guided expectations for the assembly. The NCBI assembly record reports 42× genome coverage. Hi-C sequencing produced 130.82 Gb from 433.19 million read pairs, which were used to scaffold the primary assembly. RNA sequencing data were also generated and are available in public sequence repositories. Table 1 summarises the specimen and sequencing details.

**Figure 2.**
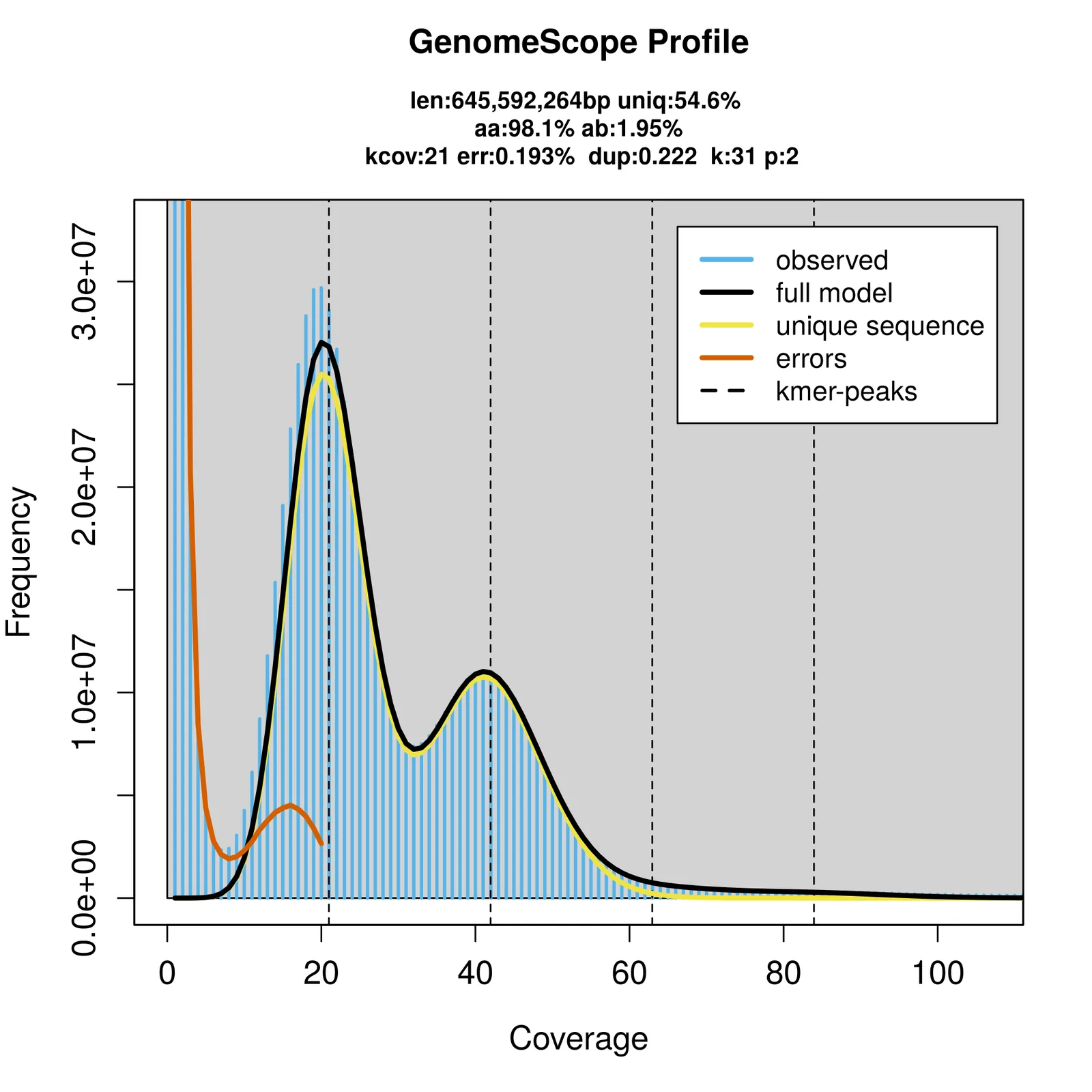
GenomeScope 2.0 model fitted to the *k*-mer frequency distribution. The plot shows the observed and modelled *k*-mer spectra used to estimate haploid genome size, heterozygosity and repeat content from unassembled sequencing reads.

**Table 1.** Specimen and sequencing data for *S. sphegea* (BioProject PRJEB59366)

**Platform**	**PacBio HiFi**	**Hi-C**	**RNA-seq**
**ToLID**	idSepSphe2	idSepSphe1	idSepSphe2
**Specimen ID**	Ox001307	NHMUK014446900	Ox001307
**BioSample (source individual)**	SAMEA10166786	SAMEA9654305	SAMEA10166786
**BioSample (tissue)**	SAMEA10201043	SAMEA9654392	SAMEA10201044
**Tissue**	head and thorax	head and thorax	abdomen
**Instrument**	Sequel II	Illumina NovaSeq 6000	Illumina NovaSeq X
**Library ID & run accession(s)**	DTOL13251668: ERR10841314	DN861909I:B2: ERR10851507	ERR13093627
**Read count total**	2.53 million reads	433.19 million read pairs	80.53 million read pairs
**Base count total**	28.84 Gb	130.82 Gb	24.32 Gb

### Assembly statistics

The primary assembly was generated and an alternate haplotype assembly was also deposited at scaffold level in INSDC databases. The primary assembly is proposed as the reference assembly for this species. It has a total length of 747.79 Mb in 166 scaffolds (excluding organelle sequences), with 654 gaps, and its scaffold N50 is 120.97 Mb (Table 2). The scaffold auN for the primary assembly is 107 Mb.

**Table 2.** Genome assembly statistics for *S. sphegea*

**Genome assembly**	**Primary assembly**	**Alternate haplotype assembly**
**Assembly name**	idSepSphe2.1	idSepSphe2.1 alternate haplotype
**Assembly accession**	GCA_988143715.1	GCA_988143865.1
**Assembly level**	chromosome	scaffold
**Span (Mb)**	747.79	548.34
**Number of chromosomes**	7	-
**Number of contigs**	820	1,478
**Contig N50 (Mb)**	2.34	1.0
**Number of scaffolds (excluding organelle sequences)**	166	541
**Scaffold N50 (Mb)**	120.97	112.93
**Scaffold auN (Mb)**	107	-
**Longest scaffold length (Mb)**	134.5	130.75
**Sex chromosomes**	X and Y	-
**Organelles**	Mitochondrial genome: 19.16 kb	-

Most of the primary assembly sequence (98.04%) was assigned to 7 chromosome-level scaffolds, representing 5 autosomes and the X and Y sex chromosomes. These chromosome-level scaffolds, confirmed by Hi-C data, are named according to size (Figure 3; Table 3). During curation, we noted that the order and orientation of scaffolds are uncertain in the following regions: chromosome 2 ~101.05–103.65 Mb, chromosome 3 ~39.47–43.08 Mb, chromosome 4 ~3.26–5.36 Mb, chromosome 5 16.88–19.99 Mb, and the entirety of chromosomes X and Y. Chromosomes X and Y were identified by their copy number in the diploid assembly, and X was assigned based on BUSCO alignments.

The mitochondrial genome was also assembled (length 19.16 kb, OZ532787.1). This sequence is included as a contig in the multifasta file of the primary genome submission and as a standalone record.

**Figure 3.**
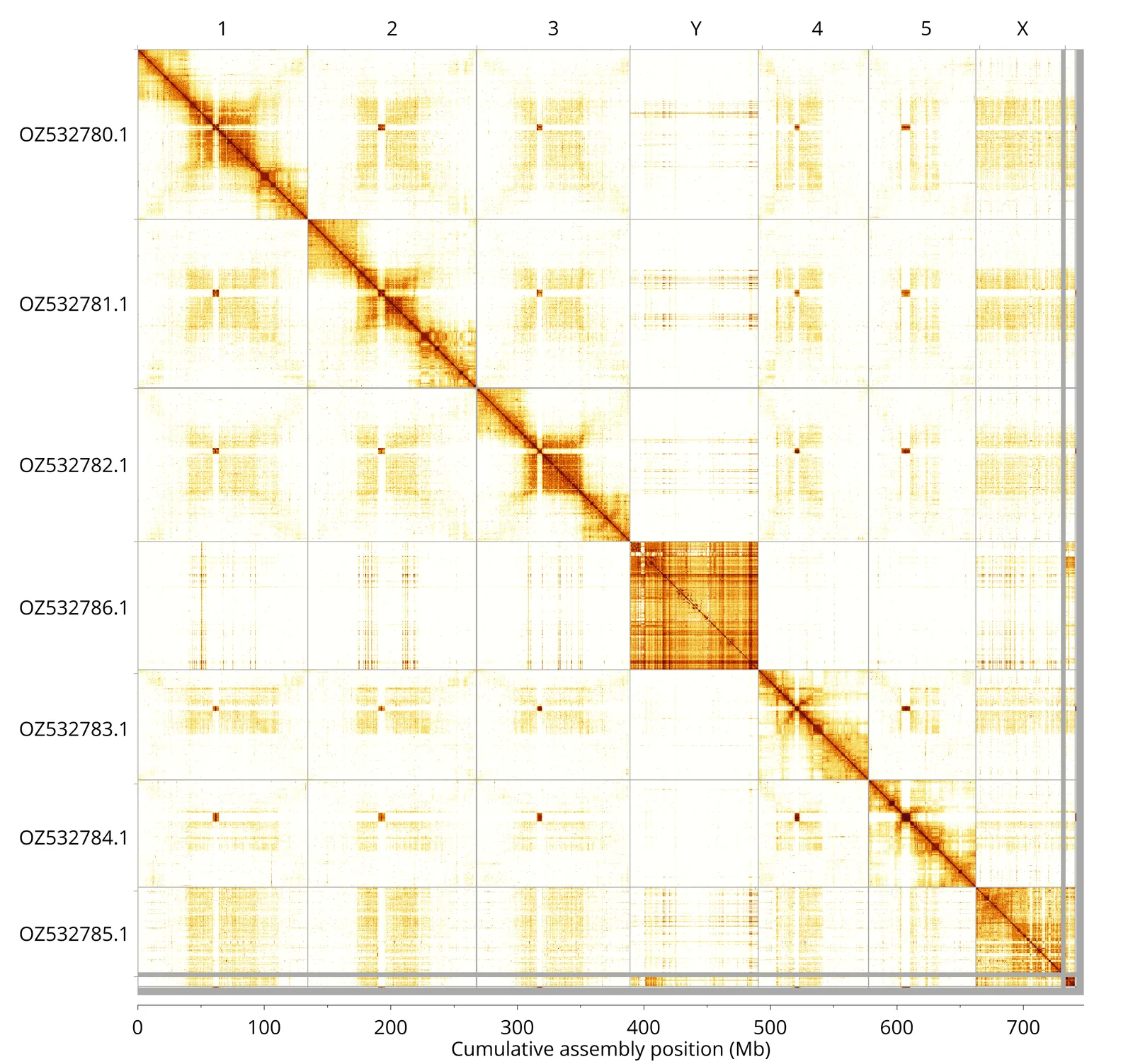
Hi-C contact map of the *S. sphegea* primary assembly. Assembled chromosomes are shown in order of size and labelled along the axes, with a megabase scale shown below. The plot was generated using PretextSnapshot.

**Table 3.** Chromosomal pseudomolecules in the primary genome assembly of *S. sphegea* idSepSphe2

**INSDC accession**	**Molecule**	**Length (Mb)**	**GC%**
OZ532780.1	1	134.50	32.50
OZ532781.1	2	133.64	32.50
OZ532782.1	3	121.12	32.50
OZ532783.1	4	87.19	32
OZ532784.1	5	84.75	32
OZ532785.1	X	67.57	34
OZ532786.1	Y	104.40	30.50

Chromosome painting shows the distribution of BUSCO genes assigned to Brachycera ancestral linkage groups across the chromosome-level scaffolds (Figure 4).

**Figure 4.**
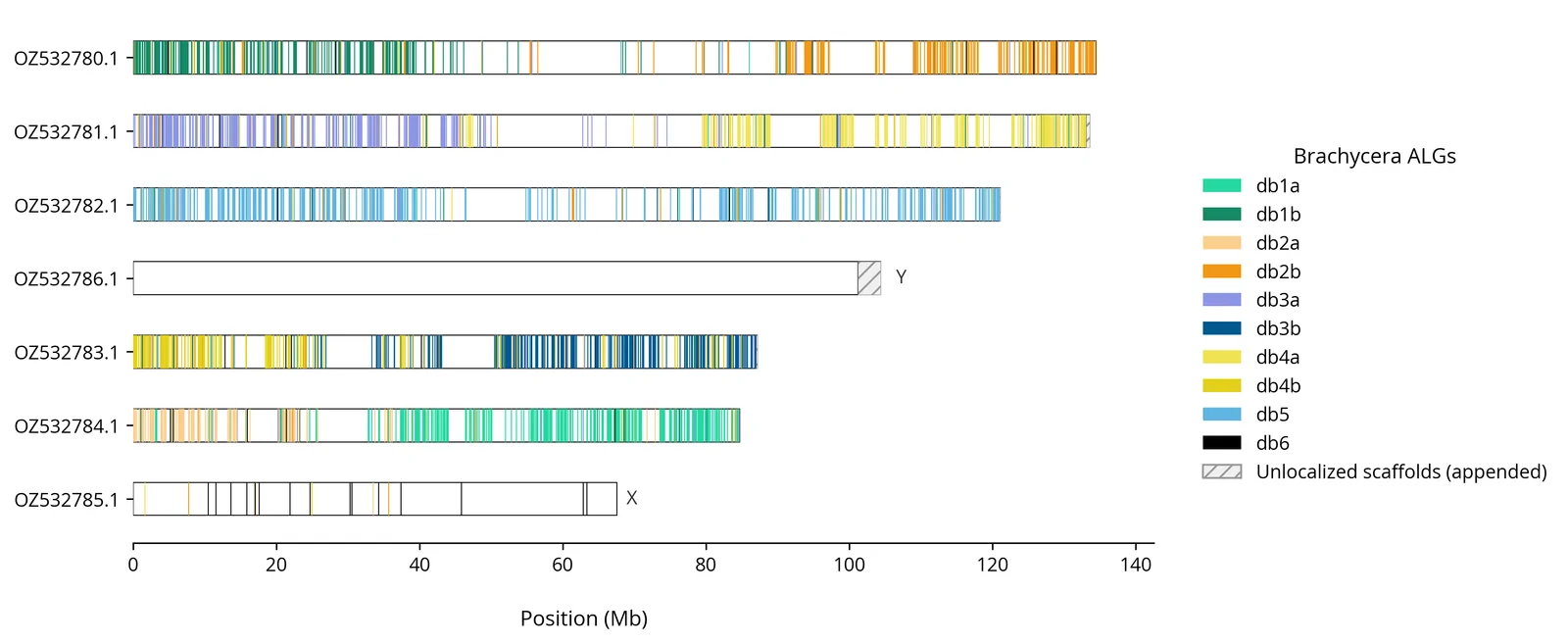
Brachycera ancestral linkage groups painting of the genome assembly of *S. sphegea*. Chromosomes are drawn to scale, with coloured bars marking the positions of complete diptera_odb12 BUSCO genes. Each BUSCO gene is coloured by its assignment to one of the 10 Brachycera ALGs (db1a–db4b, db5 and db6).

### Assembly quality metrics

The combined primary and alternate assemblies achieve an estimated QV of 63.5. The *k*-mer completeness is 70.44% for the primary assembly, 62.79% for the alternate haplotype, and 97.69% for the combined assemblies (Figure 5).

**Figure 5.**
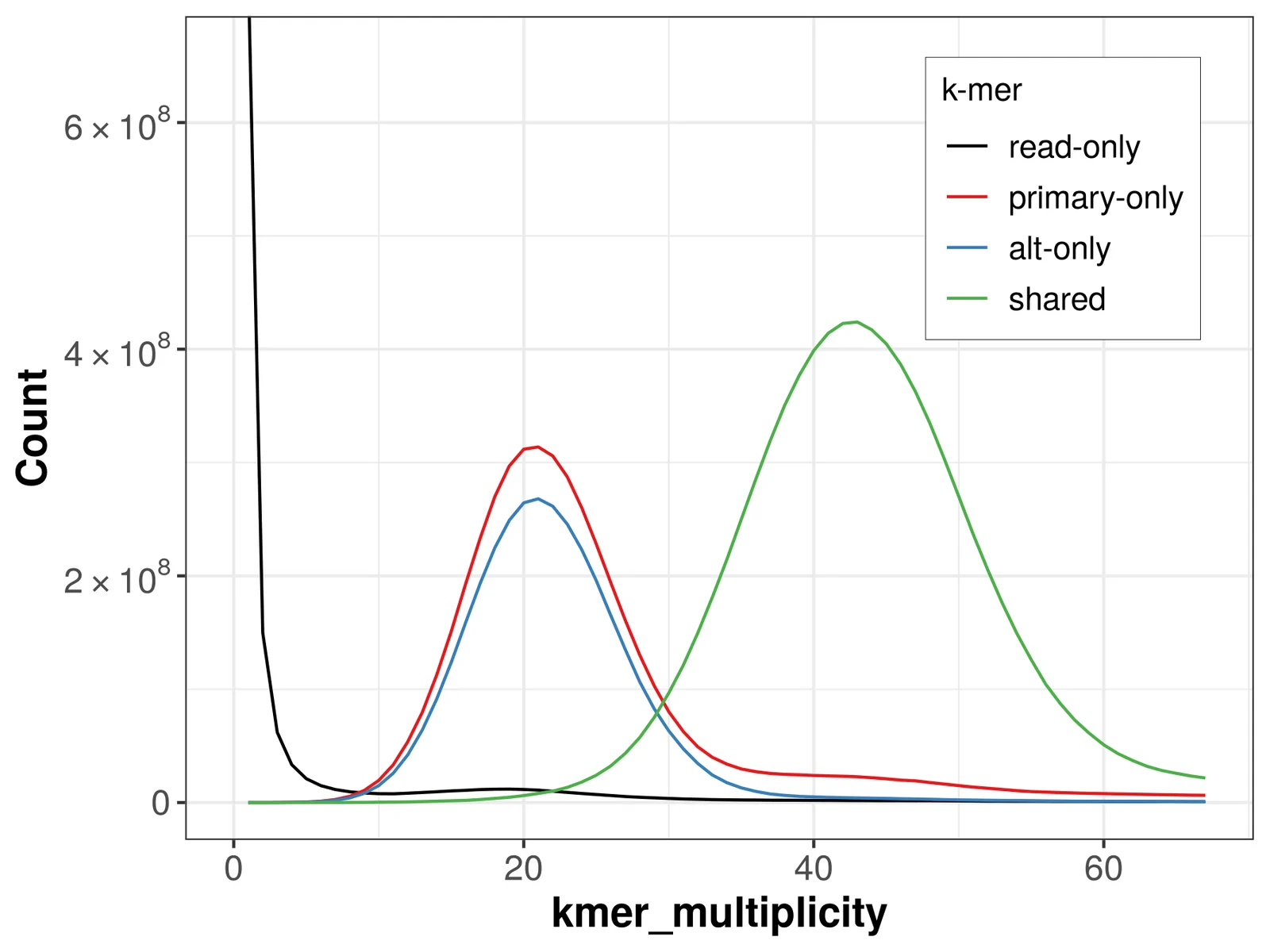
Evaluation of *k*-mer completeness using MerquryFK. This plot illustrates the recovery of *k*‐mers from the original read data in the primary assembly, the alternate haplotype assembly and the combined assemblies. The horizontal axis represents *k*‐mer multiplicity, and the vertical axis shows the number of *k*‐mers. The black curve represents *k*‐mers that appear in the reads but are not assembled. The green curve corresponds to *k*‐mers shared by both haplotypes, and the red and blue curves show *k*‐mers found only in one of the haplotypes.

BUSCO analysis of the primary assembly using the brachycera_odb12 reference set ( *n* = 5,912) identified 98.1% of the expected gene set (single = 97.2%, duplicated = 0.9%).

The snail plot in Figure 6 provides a summary of assembly contiguity, cumulative scaffold count and sequence composition for the primary assembly ( Challis & Blaxter, 2026). The relative sizes of the longest scaffold, scaffold N50 and N90, together with the dark-grey scaffold-length distribution, show how much of the assembly is contained in long scaffolds. The outer composition track shows variation in GC, AT and N content, while the central track shows the cumulative scaffold count.

The blob plot in Figure 7 displays scaffolds from the primary assembly by GC proportion and base coverage. Marker area is proportional to scaffold length, and colour indicates taxonomic assignment ( Challis *et al.*, 2020).

**Figure 6.**
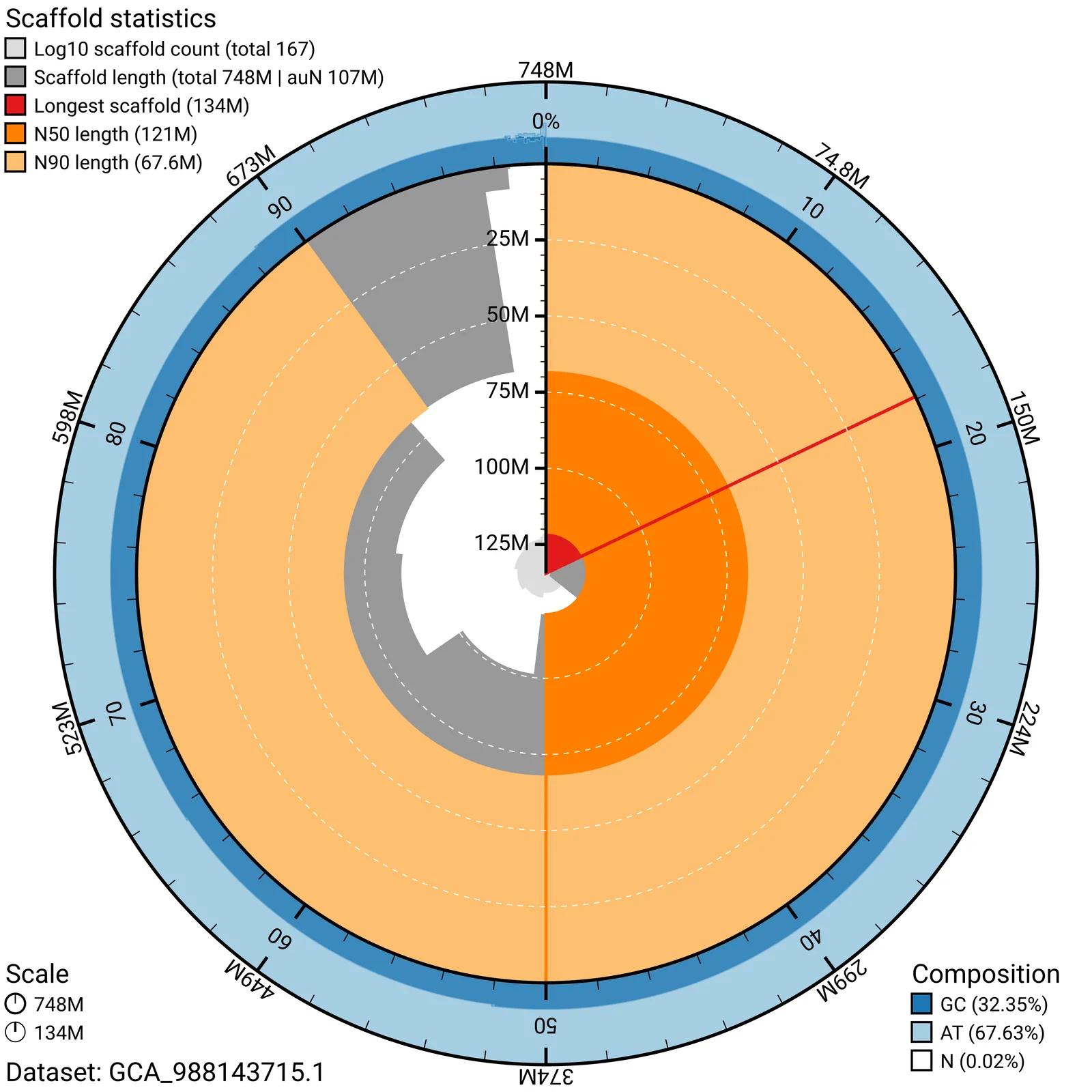
Assembly metrics for idSepSphe2.1, the primary assembly. The BlobToolKit snail plot provides an overview of assembly contiguity and sequence composition. The circumference represents the length of the primary assembly, and the main plot is divided into 1,000 bins around the circumference. The outermost blue tracks display the distribution of GC, AT, and N percentages across the bins. Scaffolds are arranged clockwise from longest to shortest and are depicted in dark grey. Scaffold length is shown on a linear radial scale, scaled to the longest scaffold. Red shading indicates the longest scaffold, and the deeper orange and pale orange shading represent the N50 and N90 lengths. The summary statistics include scaffold auN, the area under the scaffold Nx curve. The light grey spiral at the centre shows the cumulative scaffold count on a logarithmic scale. The assembly can be explored interactively in the BlobToolKit viewer.

**Figure 7.**
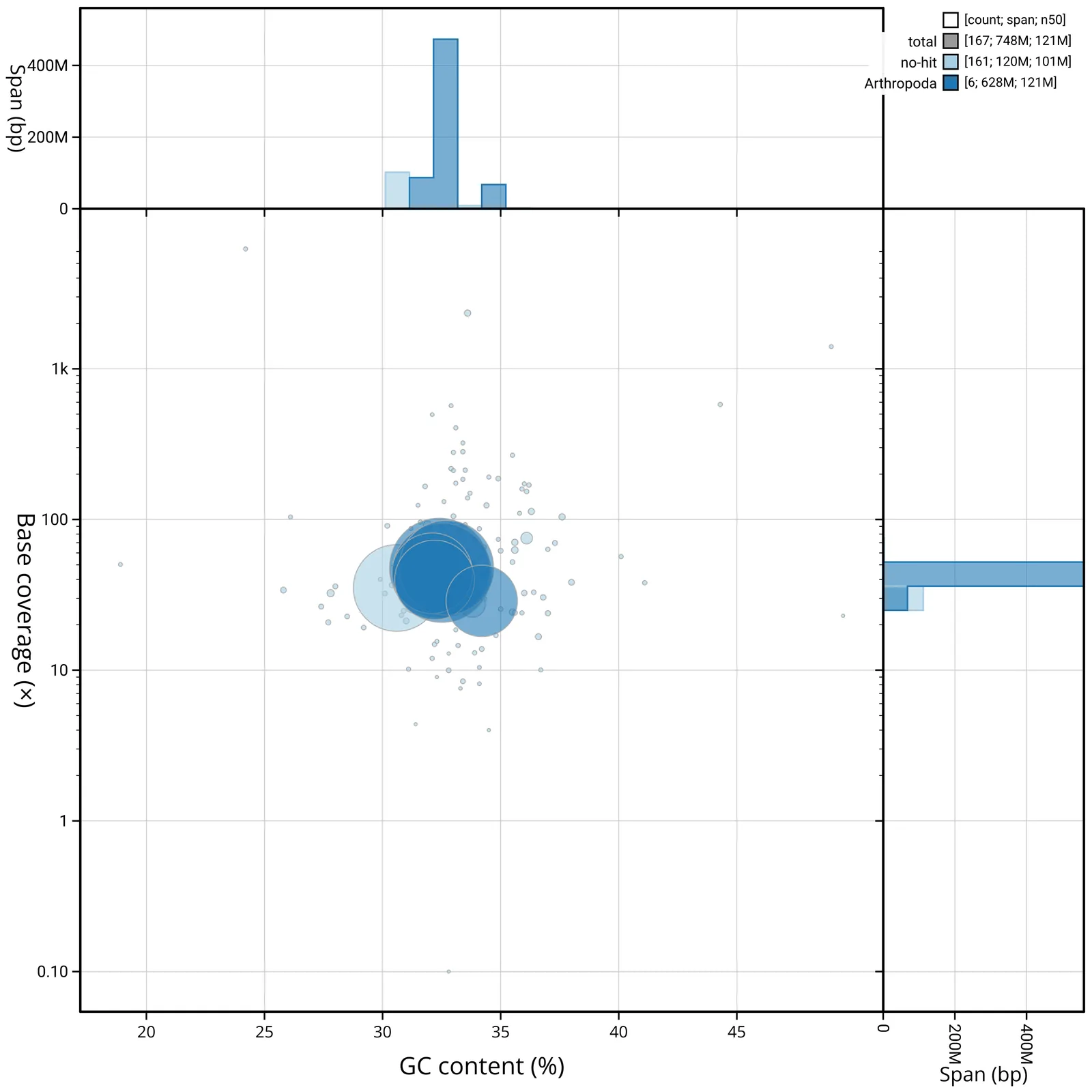
BlobToolKit blob plot for idSepSphe2.1. The plot shows base coverage (vertical axis) and GC content (horizontal axis). Circles represent scaffolds; marker area is proportional to scaffold length, and colour indicates taxonomic assignment. The histograms along the axes display the total length of sequences distributed across different levels of coverage and GC content. The assembly can be explored interactively in the BlobToolKit viewer.

Table 4 presents assembly quality metrics alongside the Earth BioGenome Project (EBP) benchmarks described by Howe *et al.*. (2026). The three-part EBP metric, calculated for the primary assembly, is **6.C.Q62**.

**Table 4.** Earth BioGenome Project summary metrics for the *S. sphegea* assembly

**Measure**	**Value**	***Benchmark***
EBP summary (primary)	6.C.Q62	*6.C.Q40*
Contig N50 length	2.34 Mb	*> 1 Mb*
Scaffold N50 length	120.97 Mb	*= chromosome N50*
Consensus quality (QV)	Primary: 62.8; alternate: 64.9; combined: 63.5	*≥ 40*
*k*-mer completeness	Primary: 70.44%; alternate: 62.79%; combined: 97.69%	*> 90% (combined assembly)*
BUSCO	C:98.1% [S:97.2%, D:0.9%], F:0.6%, M:1.3%, n:5,912	*S > 90%*
Percentage of assembly assigned to chromosomes	98.04%	*≥ 90%*

**Notes:** The EBP summary reports log10(contig N50), rounded down; C for chromosome-scale scaffolding or log10(scaffold N50), rounded down; and Q for consensus quality (Merqury QV). N50 values are in base pairs. The *k*-mer completeness benchmark applies to the combined assembly. BUSCO v6.0.0, brachycera_odb12: C=complete; S=single-copy; D=duplicated; F=fragmented; M=missing; n=orthologues. BUSCO short summary: primary assembly.

### Acknowledgements

With thanks to Oliver Poole for proofreading the Background text.

### Data availability

European Nucleotide Archive: Sepedon sphegea, idSepSphe. Accession number PRJEB59366; https://identifiers.org/ena.embl/PRJEB59366. The genome sequence is released openly for reuse. The *S. sphegea* genome sequencing initiative is part of the Darwin Tree of Life Project ( PRJEB40665) and the Sanger Institute Tree of Life Programme ( PRJEB43745). All raw sequence data and the primary and alternate assemblies have been deposited in INSDC databases. The genome will be annotated using available RNA-Seq data and presented through the Ensembl pipeline at the European Bioinformatics Institute. Raw data and assembly accession identifiers are reported in Table 1 and Table 2.

Production code used in genome assembly at the WSI Tree of Life is available at https://github.com/sanger-tol. Table 5 lists software versions used in this study.

**Table 5.** Software versions and sources

**Software**	**Version**	**Source**
BLAST	2.14.0	https://ftp.ncbi.nlm.nih.gov/blast/executables/blast+/
BlobToolKit	4.4.6	https://github.com/genomehubs/blobtoolkit
BlobTk (hosted BlobDir)	0.8.1	https://github.com/genomehubs/blobtk
BlobTk (snail and blob plot renderer)	0.8.3	https://github.com/genomehubs/blobtk
BUSCO	6.0.0	https://gitlab.com/ezlab/busco
busco-alg-painter	0.1.0	https://github.com/Karenvn/busco-alg-painter
bwa-mem2	2.2.1	https://github.com/bwa-mem2/bwa-mem2
DIAMOND	2.1.16	https://github.com/bbuchfink/diamond
fasta_windows	0.2.4	https://github.com/tolkit/fasta_windows
FastK	1.2	https://github.com/thegenemyers/FASTK
GenomeScope 2.0	2.1.0	https://github.com/tbenavi1/genomescope2.0
Gfastats	1.3.6	https://github.com/vgl-hub/gfastats
Hifiasm	0.16.1-r375	https://github.com/chhylp123/hifiasm
HiGlass	1.13.4	https://github.com/higlass/higlass
MerquryFK	1.1.0-c1	https://github.com/thegenemyers/MERQURY.FK
Minimap2	2.29-r1283	https://github.com/lh3/minimap2
miniprot	0.18-r281	https://github.com/lh3/miniprot
MitoHiFi	2	https://github.com/marcelauliano/MitoHiFi
MultiQC	1.14; 1.17 and 1.18	https://github.com/MultiQC/MultiQC
Nextflow	25.10.4	https://github.com/nextflow-io/nextflow
PretextSnapshot	0.0.6	https://github.com/sanger-tol/PretextSnapshot
PretextView	1.0.3	https://github.com/sanger-tol/PretextView
purge_dups	1.2.3	https://github.com/dfguan/purge_dups
samtools	1.23.1	https://github.com/samtools/samtools
sanger-tol/ascc	0.1.0	https://github.com/sanger-tol/ascc
sanger-tol/blobtoolkit	v0.11.1; 0.11.1	https://github.com/sanger-tol/blobtoolkit
sanger-tol/curationpretext	1.4.2	https://github.com/sanger-tol/curationpretext
Seqtk	1.4-r122	https://github.com/lh3/seqtk
Singularity	3.9.0	https://github.com/sylabs/singularity
TreeVal	1.4.0	https://github.com/sanger-tol/treeval
YaHS	yahs-1.2a.1	https://github.com/c-zhou/yahs

### Author information

Contributors are listed at the following links:

- Members of the University of Oxford and Wytham Woods Genome Acquisition Lab
- Members of the Natural History Museum Genome Acquisition Lab
- Members of the Darwin Tree of Life Barcoding collective
- Members of the Wellcome Sanger Institute Tree of Life Management, Samples and Laboratory team
- Members of Wellcome Sanger Institute Scientific Operations – Sequencing Operations
- Members of the Wellcome Sanger Institute Tree of Life Core Informatics team
- Members of the Tree of Life Core Informatics collective
- Members of the Darwin Tree of Life Consortium

### Wellcome Sanger Institute – Legal and Governance

The materials that have contributed to this genome note have been supplied by a Darwin Tree of Life Partner. The submission of materials by a Darwin Tree of Life Partner is subject to the **‘Darwin Tree of Life Project Sampling Code of Practice’**, which can be found in full on the Darwin Tree of Life website. By agreeing with and signing up to the Sampling Code of Practice, the Darwin Tree of Life Partner agrees they will meet the legal and ethical requirements and standards set out within this document in respect of all samples acquired for, and supplied to, the Darwin Tree of Life Project. Further, the Wellcome Sanger Institute employs a process whereby due diligence is carried out proportionate to the nature of the materials themselves, and the circumstances under which they have been/are to be collected and provided for use. The purpose of this is to address and mitigate any potential legal and/or ethical implications of receipt and use of the materials as part of the research project, and to ensure that in doing so we align with best practice wherever possible. The overarching areas of consideration are:

- Ethical review of provenance and sourcing of the material
- Legality of collection, transfer and use (national and international)

Each transfer of samples is further undertaken according to a Research Collaboration Agreement or Material Transfer Agreement entered into by the Darwin Tree of Life Partner, Genome Research Limited (operating as the Wellcome Sanger Institute), and in some circumstances, other Darwin Tree of Life collaborators.
