# Supplementary material for: The genome sequence of the Blue Long-horned Snailkiller, Sepedon sphegea (Fabricius, 1775) (Diptera: Sciomyzidae)
Source: Wellcome Open Res. 2026 Oct 28;11:937. [Version 1] doi: 10.12688/wellcomeopenres.28240.1 (PMC13743838; doi:10.12688/wellcomeopenres.28240.1)
Supplement: Peer review history

# Reviewer report: Reviewer response for version 1

**DOI:** 10.21956/wellcomeopenres.31068.r177102

**Published:** 2026-11-06

**Authors:** François Renoz

**Affiliations:** 1 UC Louvain, Louvain, Belgium

I have carefully read the article by Falk et al. It is a genome report. The quality of the assembly is very good: 98.04% of the sequence is assigned to 7 chromosomes, the BUSCO score is 98.1% (D = 0.9%), the combined completeness of the k-mers is 97.7%, and the QV is 63.5. The N50 contig length of 2.34 Mb is acceptable, though not exceptional. Public accessions, software versions, and pipelines are well documented. Overall, these values are very good and I have no “super” major comments to make. But additional information would make the results more accurate and the article clearer and more appealing. Here are my comments:

 (Gaponov, 2016; Neff & Berg, 1966): UK-specific statements (overwintering adults, March-October flight period in the UK) are supported by a Russian (Gaponov 2016) and US (Neff & Berg 1966) studies. Please cite UK sources if possible.

 Paragraph “We present a chromosome-level genome sequence for *Sepedon sphegea*, produced using the Tree of Life pipeline…”: please, Suggest 1-2 sentences on the specific value of the assembly.

 Paragraph “Two adult *Sepedon sphegea *specimens were used: a male specimen for genome and RNA sequencing (specimen ID Ox001307, ToLID idSepSphe2; Figure 1) and a specimen for Hi-C sequencing (specimen ID NHMUK014446900, ToLID idSepSphe1).”: Hi-C was generated from a different individual (idSepSphe1, different site) than the HiFi data. Its sex (we assume it is also a male, but it is not clear.), collection date and life stage are not given. A female Hi-C library would provide no information on Y, and scaffolding of X and Y with a different individual is not justified. Please (i) state the sex of idSepSphe1, (ii) discuss possible structural variation between the two individuals, (iii) state how this affects confidence in X and Y scaffolding.

 Mitochondrial genome (19.16 kb) is reported without annotation (gene content/order), circularity, or comparison with the reference used (Trypetoptera punctulata, MK644823.1). Please, provide more information.

 “The assembly was decontaminated using the Assembly Screen for Cobionts and Contaminants (ASCC) pipeline.”: ASCC is mentioned but no result is reported: number/size of scaffolds removed, taxonomic nature of contaminants/cobionts, and whether any symbiont or parasite sequences were found. Fig. 7 shows 161 “no-hit” scaffolds, without explanation of their composition. Please, provide additional information.

 Y (104.40 Mb) is larger than X (67.57 Mb) and has no BUSCO genes (Fig. 4). This unusually large Y is not discussed. Please comment on its likely repeat content, whether it may include collapsed/misassembled sequence, and whether Hi-C contacts support it as a single chromosome (Fig. 3 shows a dense but diffuse block).

 Fig. 4 is presented without interpretation. Please, provide more information, in particular for X and Y chromosomes (few or no BUSCO genes).  

 Fig. 7 legend: 161 of 167 scaffolds (120 Mb) have no taxonomic hit, and only 6 scaffolds (628 Mb) are assigned to Arthropoda. Please clarify which chromosomes fall in the no-hit category (presumably Y and small scaffolds).

 Hifiasm 0.16.1 is quite an older release (2021). Please justify its use (pipeline consistency). Why not use a more recent version?

Are the datasets clearly presented in a usable and accessible format, and the assembly and annotation available in an appropriate subject-specific repository?

Yes

Are sufficient details of the sequencing and extraction, software used, and materials provided to allow replication by others?

Partly

Are the rationale for sequencing the genome and the species significance clearly described?

Partly

Are the protocols appropriate and is the work technically sound?

Yes

Reviewer Expertise:

Evolution and Functioning of Bacterial Symbiosis in Insects

I confirm that I have read this submission and believe that I have an appropriate level of expertise to confirm that it is of an acceptable scientific standard.

# Reviewer report: Reviewer response for version 1

**Published:** 2026-10-28

**Authors:** Automated Benchmarking

**Affiliations:** 1 Wellcome Sanger Institute, Hinxton, UK

As this article is a Genome Note, Automated Benchmarking has been used to determine whether the reported assembly passes four defined checks established by the Earth BioGenome Project: the raw and assembled data are publicly available, the Earth BioGenome Project three-part metric is met, the k-mer completeness exceeds 90%, and the BUSCO score passes the stated threshold. For Sepedon sphegea (idSepSphe2), all four checks passed. Data available: pass. ENA study PRJEB59366, raw-read accessions ERR10841314, ERR10851507, and ERR13093627, and assembly accessions GCA_988143715.1 and GCA_988143865.1 are public. Earth BioGenome Project three-part metric: pass. The primary assembly is reported as 6.C.Q62, meeting the 6.C.Q40 reference standard. k-mer completeness: pass. The combined haplotype assemblies have 97.69% k-mer completeness, exceeding the Earth BioGenome Project target of 90%. BUSCO: pass. The primary assembly, which is proposed as the reference assembly for this species, has 98.1% complete BUSCOs (97.2% single-copy and 0.9% duplicated). BUSCO software version: 6.0.0; lineage dataset: brachycera_odb12. It meets the target of greater than 90% complete and single-copy BUSCOs. This decision uses values reported in the Genome Note and public accession records.

Reviewer Expertise:

NA

I confirm that I have read this submission and believe that I have an appropriate level of expertise to confirm that it is of an acceptable scientific standard.
